# Research on the Performance of Self-Made Open-Cathode Fuel Cell Stacks under Different Operating Conditions

**DOI:** 10.3390/membranes13110881

**Published:** 2023-11-13

**Authors:** Qiang Bai, Zhenghong Liu, Chuangyu Hsieh

**Affiliations:** 1 School of Mechanical Engineering, Guiyang University, Guiyang 550002, China; baiqiang@gyu.cn; 2 Department of Automotive Engineering, Tsinghua University, Beijing 100084, China; heyx19@mails.tsinghua.edu.cn

**Keywords:** PEMFC, open cathode, low humidity

## Abstract

The traditional fuel cell power system requires external ventilation and humidification systems for both the anode and cathode, which not only increases the application cost but also restrict its widespread use. In order to further enhance the applicability and reduce the operating costs of fuel cell power systems, this paper investigates the open-cathode proton exchange membrane fuel cell power system. This approach not only lowers the cost but also reduces the weight of the power system, enabling its potential application in a wider range of vehicles. In this study, two versions of the open-cathode fuel cell stacks were developed and performance and stability tests were conducted under various operating conditions. Additionally, tests were carried out with different materials of carbon paper to find a balance between performance and stability. Through the research presented in this paper, the application scope of fuel cells has been expanded, providing valuable insights for their further development.

## 1. Introduction

Whether it is a lithium cell with a history of over half a century [1,2,3] or a fuel cell with a history of over a century [4,5,6], the mechanism of a fuel cell has been unknown since its discovery. Nevertheless, with the gradual maturation of technology, lithium cells found widespread adoption in the early 21st century as a power source for mobile phones. Furthermore, in the context of the burgeoning new energy vehicle market, lithium fuel cells have maintained their significance in the field of transportation. In stark contrast, fuel cells have appeared to linger in relative obscurity, primarily owing to their higher production costs and increased system complexity. Nonetheless, it is imperative to recognize that fuel cells possess distinctive advantages, such as rapid refueling capabilities and the elimination of range anxiety, differentiating them significantly from their lithium counterparts. These attributes have propelled fuel cells into the focal point of extensive global research efforts. Lei Jilin et al. [7] studied the performance change of open-cathode proton exchange membrane fuel cells in a dry environment and provided guidance for the design of bipolar plate (BP) graphite in OC-PEMFC. Zipeng Huang et al. [8] studied the performance of air-cooled fuel cell stacks after integrating Vapor Chambers (VCs). The integration of VCs effectively improved the thermal management of the stack and improved fuel cell performance. Xingzi Yu et al. [9] studied the performance changes of the stack under different fan powers and found that the fuel cell can achieve the best performance when the fan works at an operating voltage of 8.5 V. Jikai Zhang et al. [10] studied the temperature and performance changes of an open-cathode fuel cell stack in a thermal radiation environment. From the results, it can be seen that although the thermal radiation environment is conducive to increasing the operating temperature of the stack, the performance is not good due to insufficient cooling capacity. Zixuan Wang et al. [11] studied the performance changes of fuel cells under different cathode flow fields and found that the metal foam flow field has the best performance and that temperature and wind speed need to be controlled within a certain range to make the fuel cell perform optimally. Xianxian Yu et al. [12] designed a new bipolar plate flow channel—concave–convex dual-flow-channel bipolar plates. This method not only improves the thermal management efficiency of the stack but also makes the internal temperature of the fuel cell more uniform. Kehan Zhou et al. [13] designed an air-cooled fuel cell stack with an inclined cathode flow structure for unmanned aerial vehicles, which not only improves fuel cell performance but also reduces its weight and volume.

Lu Liu et al. [14] studied an open-cathode PEM fuel cell temperature dynamic model based on the online T-S fuzzy identification method. This method not only has the advantages of low computational cost and low model parameter requirements but is also one of the most promising methods for identifying dynamic models in a control system. Shanal S Kumar et al. [15] proposed a simplified nonlinear model for open-cathode PEMFC and controlled it using two different control strategies. The model’s prediction results closely matched experimental data, making it suitable for early design research in PEMFC systems. Joseph Ishaku et al. [16] established control-oriented nonlinear models for various components of the open-cathode fuel cell power system, capturing the dynamics and interactions between them. Yakoub Zine et al. [17] addressed the significant parameter variations in the open-cathode fuel cell power system by designing and studying a fuzzy logic controller to track the maximum efficiency point region, demonstrating the effectiveness of this method through experiments. Chaima Mahjoubi et al. [18] proposed an improved control strategy for open-cathode proton exchange membrane fuel cells, where a single actuator controls both air supply and stack temperature, ensuring the high efficiency and long life of the fuel cell stack. Mohsen Kandidayeni et al. [19] introduced a system management design to enhance the energy efficiency of open-cathode proton exchange membrane fuel cells (PEMFCs) in hybrid systems. From the literature review above, it is evident that current research on open-cathode fuel cells primarily focuses on improving cell performance and power control systems. Cell performance is fundamental in determining the overall system performance, so this paper will approach open-cathode fuel cell research from the perspective of cell performance.

To address the challenges of fuel cell humidification and complex water management systems, this paper will focus on studying the performance changes of fuel cell stacks under low humidification conditions, contributing to the commercialization of fuel cells. This paper aims to explore the performance variations of an open-cathode proton exchange membrane fuel cell (PEMFC) under different humidity conditions, and based on these performance changes, make improvements to the membrane electrode assembly (MEA). The specific research objectives are as follows: (1) investigate the structure of the open-cathode PEMFC and independently fabricate the fuel cell; (2) study the effects of varying he operating conditions and gas quantities on cell humidity and performance; (3) enhance the materials of the MEA according to the observed performance changes to subsequently elevate cell performance; and (4) re-test the improved fuel cell and conduct a comparative analysis with existing research results.

## 2. Materials and Methods

**Figure 1:**
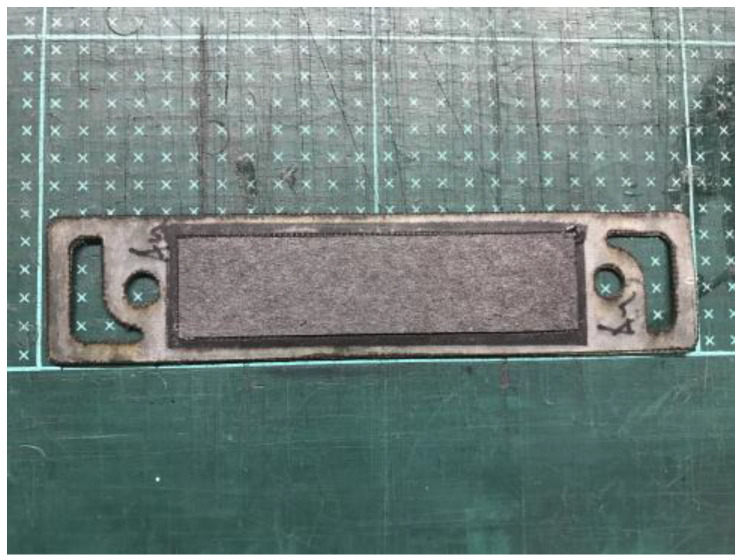
Single open-cathode fuel cell.

**Figure 2:**
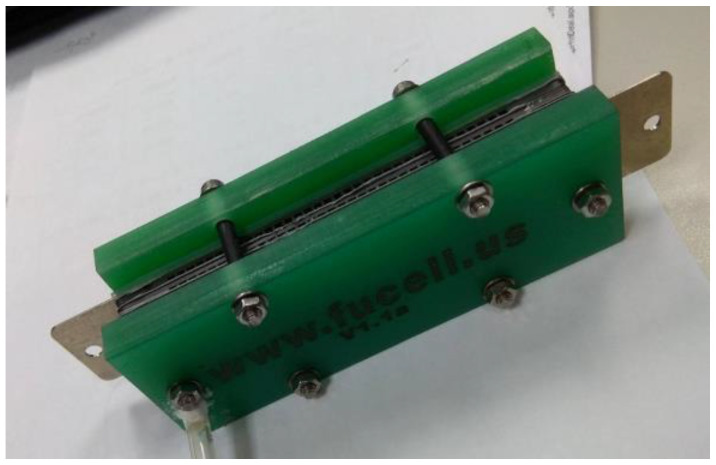
Open-cathode fuel cell stacks.

The fabrication of fuel cells is the first step in conducting this research. In this study, a single cell with an area of 9 cm^2^ (as shown in Figure 1) and an open-cathode short stack of three cells with a total reaction area of 21.56 cm^2^ (as shown in Figure 2) were made.

### 2.1. Fuel Cell Production

(a) Thin-film electrode:

This study endeavors to examine the influence of gas diffusion layers on proton exchange membrane fuel cells operating under low-humidity conditions. To circumvent the direct application of a catalyst onto the gas diffusion layers and the ensuing ramifications on their operational efficiency, this research adopts a catalyst-coated membrane (CCM) methodology. Within this framework, the catalyst is precisely deposited onto the membrane material through a spray-coating process, thereby forming thin electrodes.

(b) Gas diffusion layer:

The gas diffusion layer serves as a crucial component within the context of fuel cell technology, and its primary role is to leverage its internal porous architecture for the swift and uniform diffusion of gases as they ingress into the fuel cell’s interior. This essential function aims to optimize the chemical reaction occurring on the catalyst layer. Furthermore, the gas diffusion layer is required to exhibit exceptional electronic conductivity to efficiently eliminate the liquid water that arises at the cathode end due to electrochemical processes. In essence, the gas diffusion layer must proficiently carry out a set of vital functions, which are typically delineated as follows:

(1) Reactant Permeability: allowing reactants to smoothly reach the catalytic layer for electrochemical reactions.
(2) Product Permeability: Providing a pathway for the discharge of generated water to prevent blocking the transfer of reaction gases.
(3) Conductivity: Transmitting the electrons generated at the catalytic layer to the current collector.
(4) Mechanical Strength: Endowing the membrane electrode assembly with a certain level of mechanical strength.
(5) Thermal Conductivity: Conducting away the heat generated by the electrochemical reactions in the membrane electrode assembly.

Since this study will be conducted under low humidification conditions, hydrophilic silicon dioxide will be added to the MEA as a control group.

(c) Leak-proof gasket:

When assembling fuel cells, a recurrent challenge arises in the form of gas leakage occurring between the graphite flow plate and the membrane electrode assembly (MEA). To mitigate this issue and ensure a gas-tight seal, a leak-proof gasket is systematically employed at this interface. The prevailing choice of leak-proof material for individual cells is polytetrafluoroethylene (PTFE), while silicone rubber has an application in the context of cell stacks.

Maintaining precise pressure conditions within the cell is of paramount importance. Excessive internal pressure can impede the ingress of gas into the membrane electrode assembly, consequently leading to a reduction in the limiting current. Conversely, inadequate pressure can result in an escalation of the ohmic impedance. Consequently, the incorporation of PTFE and silicone rubber leak-proof gaskets is instrumental in regulating compression and effectively addressing concerns pertaining to gas tightness.

(d) Graphite flow channel plate:

The primary function of the graphite flow channel plate lies in its pivotal role of directing gas ingress, product egress, and electron conduction within the fuel cell system. As such, it is imperative that the material employed for constructing the flow channel plate exhibits exceptional air tightness. Furthermore, given its responsibility for guiding the electrons generated during electrochemical reactions, the flow channel plate must also demonstrate superior conductivity. Ensuring uniform electrochemical reactions among gases within the fuel cell necessitates the even distribution of the flow channel plate along the contact surface of the gas diffusion layer.

Furthermore, in order to maintain consistent fuel cell temperatures and prevent material degradation resulting from uneven temperature distributions, the flow channel plate must possess robust thermal conductivity. This characteristic plays a vital role in optimizing fuel cell performance. Various configurations of flow channel plates are commonly employed, including serpentine channels, grid channels, chessboard-style channels, and bifurcated channels. Notably, in the context of this experiment, both individual cells and the entire fuel cell stack are equipped with the prevailing serpentine flow channel design, which is widely adopted in the current mainstream research and development of fuel cell technology.

(e) Metal collector plate:

The main function of the collector plate is to gather electric current and conduct the electrons generated at the electrodes to the external load. Therefore, the collector plate must have good conductivity. This experiment uses red copper as the basic material and deposits gold on the surface to prevent corrosion of the collector board during fuel cell operation.

(f) Membrane electrode assembly (MEA): The membrane electrode assembly is one of the core components of a fuel cell. Its function involves electrochemical reactions with the gases supplied to the anode and cathode. The MEA consists of three main parts: the proton exchange membrane (PEM), the catalyst layer, and the gas diffusion layer. As the proton exchange membrane fuel cell used in this paper is of the commercially available low-temperature type with a thickness of 15 μm, increasing the reactivity of the reaction gases is most effectively achieved by enhancing platinum loading. However, this approach is not in line with current development trends due to its high cost. Therefore, this paper employs nanotechnology to convert platinum into nanoparticles, thereby enhancing catalyst utilization. The platinum nanoparticles are then sprayed onto a carbon substrate to form the catalyst layer. The catalyst is sprayed onto the carbon substrate using an ultrasonic oscillation method, with an anodic spraying amount of 20 wt% and a cathodic spraying amount of 40 wt%. Consequently, the gas diffusion layer is the first component that the reaction gases come into contact with. Gases are evenly distributed in this layer to improve reaction rates and reduce activation energy as they are directed towards the catalyst layer. This leads to increased efficiency in the conversion of chemical energy into electrical energy. The gas diffusion layer used in this paper is commercially available, with a thickness of 2.4 mm.

(g) End plate:

The end plate plays a critical role in ensuring a uniform pressure distribution across the entire fuel cell, facilitating its attainment of a stable operational state during electrochemical reactions. In the course of this experimental setup, both the individual cells and the cell stack are constructed using glass fiber material. Specifically, grooves are meticulously machined into the end plate at the fuel inlet, while O-Rings are strategically positioned to effectively mitigate gas leakage from the fuel cell. Subsequently, following the secure fastening of the fuel cell using screws, the assembly of both the individual cells and the stack is considered finalized.

**Figure 3:**
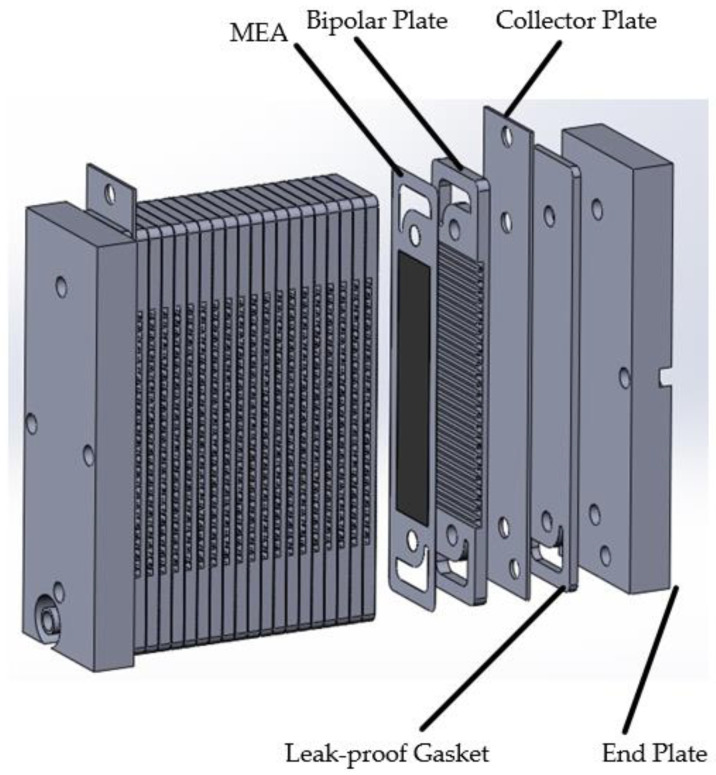
Overall architecture of open-cathode fuel cells.

Figure 3 shows the explosion diagram of the overall structure of the fuel cell, indicating that the overall structure of an open-cathode fuel cell is similar to that of a regular fuel cell.

### 2.2. Processing of Gas Diffusion Layer

To enhance the water management capabilities of the gas diffusion layer, conventional methodologies typically encompass hydrophobic treatment and the incorporation of a micro-porous layer. In the context of this investigation, an autonomous manufacturing approach for gas diffusion layers has been devised. Unlike conventional methods that are constrained by predefined specifications of commercially accessible products, this novel technique empowers the creation of bespoke gas diffusion layers tailored to the precise specifications dictated by this research endeavor.

(a) Preparation of microporous layer (MPL):

Due to the relatively low surface smoothness of some gas diffusion layer substrates, in order to enhance their surface smoothness, improve their pore structure, and increase their conductivity, a layer of conductive carbon powder is often sprayed onto the surface of the substrate material. This layer is known as the microporous layer (MPL). The introduction of an MPL helps to improve the uniformity of gas distribution inside fuel cells, which not only improves performance but also increases water production, thereby improving the internal wetness of the fuel cell. On the other hand, an increase in wetness can reduce the dryness of the MEA, thereby improving fuel cell life. The production steps are as follows:

(1) Weigh the carbon paper using a precision balance;
(2) Mix the carbon paper and isopropanol using an ultrasonic oscillation machine;
(3) Slowly add PTFE solution during the oscillation process to create a hydrophobic layer slurry of mixed carbon powder and PTFE. Simultaneously, slowly add silica powder to produce a hydrophilic layer slurry of mixed carbon powder and silica.
(4) Load the prepared mixed slurry into a spray machine and spray it onto the surface of the carbon paper.
(5) Place the sprayed carbon paper in a high-temperature sintering furnace with the following temperature settings: 120 °C for 30 min, 280 °C for 30 min, and 390 °C for 30 min.
(6) Weigh the sintered carbon paper, subtract the weight from the first step, and then divide by the area of the carbon paper to determine the content of microporous carbon powder in the gas diffusion layer.

### 2.3. Assembly of Fuel Cell Stacks

After the completion of manufacturing each component, they need to be assembled into a fuel cell stack. The specific assembly process is as follows:

(1) Wipe the components with alcohol to remove surface impurities.
(2) Cut an appropriately sized heat-shrinkable film and insert it into the screws to ensure that the fuel cell will not short-circuit.
(3) Attach four screws wrapped with insulated heat-shrinkable film to the screw caps, and then insert them into the holes on the end plate for positioning.
(4) Use a glue applicator to apply grey adhesive to the cathode and anode channels, and then assemble the bipolar plates using screws.
(5) After the membrane electrode assembly is completed, use the positions of the channel holes and screw holes to align and assemble the membrane electrode assembly.
(6) Remove the bipolar plates, overlap the gas channel reaction surface with the proton exchange membrane’s reaction area, and stack them together once the positioning is confirmed.
(7) Repeat steps (4) to (6) to complete the assembly of bipolar plates until the required number of cells, as stated in this document, is achieved.
(8) Sealing gaskets are required between the bipolar plates and the end plates to ensure sealing and assembly.
(9) Install the end plate onto the bipolar plates, place rigid spacers, and finally tighten the screw caps to ensure that pressure can be maintained for an extended period, reducing the risk of stress relaxation due to thermal expansion and contraction.
(10) Gradually increase the fastening force to 40 kgf/cm using a torque wrench in a diagonal tightening pattern.

### 2.4. Fuel Cell Gas Leakage Inspection

Air leakage constitutes a major factor contributing to the performance deterioration of fuel cells. It can be primarily categorized into two scenarios: firstly, external leakage, which can be attributed to component misalignment during assembly or inherent material defects; secondly, internal leakage occurring within the fuel cell. The origins of internal leakage mirror those of external leakage, differing only in terms of the location of misalignment or material defects as they manifest within the fuel cell itself.

Air leakage inspection primarily involves two steps: initially conducting a water droplet test for external leakage using deionized water, followed by an internal leakage test using a digital pressure gauge.

Drip leakage testing procedure:

(1) Turn on the fuel cell testing platform and introduce a certain flow of gas.
(2) Wipe the beaker with ethanol and then add a small amount of deionized water.
(3) After cleaning the dropper with deionized water, use the dropper to draw a small amount of deionized water and drop it at the edge of the bipolar plate. Observe whether there is any bubbling phenomenon.
(4) Repeat step (3) until there is no bubbling at the edges of any of the bipolar plates.
(5) If no external leakage occurs, proceed with the internal leakage test.

Leakage testing procedure:

(1) Install a gas pressure gauge behind the gas valve at the fuel cell stack inlet.
(2) After starting the fuel cell workstation, switch the anode gas to nitrogen.
(3) Use nitrogen to completely purge any residual gas inside the fuel cell.
(4) After stopping the nitrogen input, verify that the gas pressure gauge reading is zero.
(5) Close the gas outlet valve.
(6) Introduce nitrogen until the reading on the gas pressure gauge rises to 1 kg/cm^2^.
(7) Close the gas inlet valve.
(8) Observe if there is any change in the pressure gauge reading. If there is no change, it indicates that there is no gas leakage inside the fuel cell. Otherwise, it suggests the presence of a gas leakage.

At this point, the fuel cells used in this study have been manufactured and tested. After installing the assembled fuel cell stack on the testing workstation, the research testing can commence.

## 3. Results

### 3.1. Laboratory Instrument

**Figure 4:**
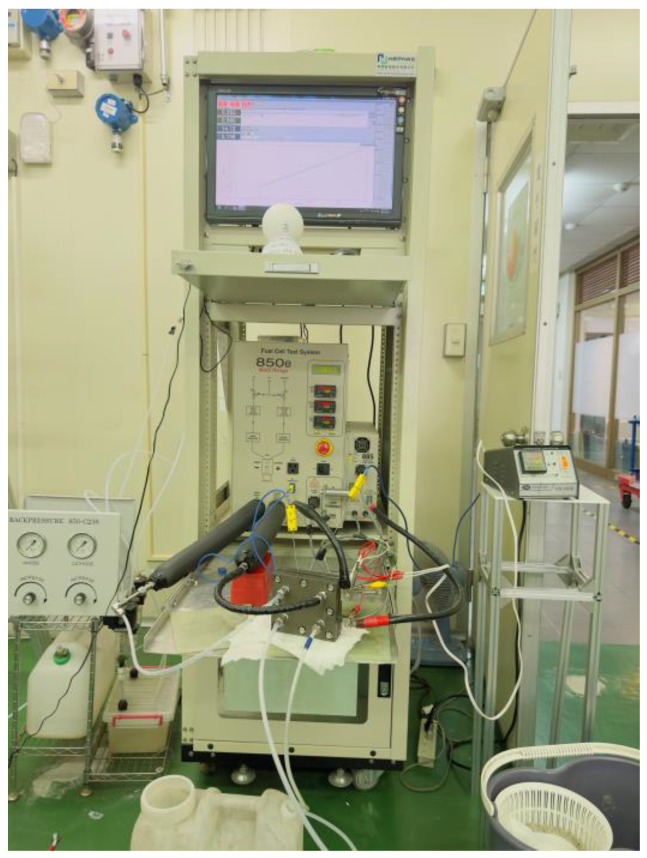
Cell testing platform.

The experiment employs a fuel cell testing system manufactured by Scribner Associates (illustrated in Figure 4), designated as model number 850 C. Initially, the fuel cell is activated, and subsequently, a performance curve of the cell is generated. AC impedance and cyclic voltammetry (CV) measurements for the catalyst electrochemical analysis are carried out.

The experimental procedure is as follows:

(1) Assemble the fuel cell and confirm the fuel cell’s tightness. Check for any gas leakage or short-circuit situations.
(2) Install the fuel cell on the testing machine and activate the gas reaction switch.
(3) Verify the water level in the humidification bottle of the testing machine.
(4) Turn on the testing machine and the status monitoring software.
(5) Set up the experimental parameters and initiate the test once the fuel cell temperature and humidifier reach the designated values.

### 3.2. Configuration of Test Parameters

The objective of configuring parameters is to replicate the operational conditions of fuel cells within real-world application scenarios. The parameters encompassed within this section consist of the anode electrochemical stoichiometry, anode humidification temperature, extended discharge duration, alternating current impedance testing, and humidification testing.

Setting of parameters for the first version of the fuel cell stack:

The anode is compared for performance using flow rates of 1500 mL/min and 1300 mL/min, with the anode being fully humidified at 100%. Forced convection is applied to the cathode section using a fan. The minimum flow rate for the cathode is:

1 A/cm^2^ × 20 cell × 9(cm^2^/cell) × 7(Anode Conversion Ratio Value) = 1260 mL/min (1)

Setting of parameters for the second version of the fuel cell stack:

The anode is operated at a flow rate of 600 mL/min, while the cathode is operated at a flow rate of 2500 mL/min. After both the anode and cathode have been humidified to 100% and activated separately, performance testing is conducted under low-humidity conditions. The minimum flow rate for the cathode is

1 A/cm^2^ × 3 cell × 21.56(cm^2^/cell) × 7(Anode Conversion Ratio Value) = 452.76 mL/min (2)

### 3.3. Results and Discussion

This experiment employs a custom-made fuel cell stack. Given the significant production of liquid water and heat during the operation of the fuel cell, it is imperative to perform a series of tests on the fuel cell under various operational conditions post-assembly. This approach facilitates an in-depth exploration of the fuel cell’s internal state alterations. In the context of this research, we utilize the 850 °C platform to replicate the influence of diverse operating conditions on the performance of the fuel cell. We undertake a performance analysis to establish a theoretical framework and amass empirical insights to advance the development of open-cathode fuel cells.

#### 3.3.1. First Version Performance Analysis

**Figure 5:**
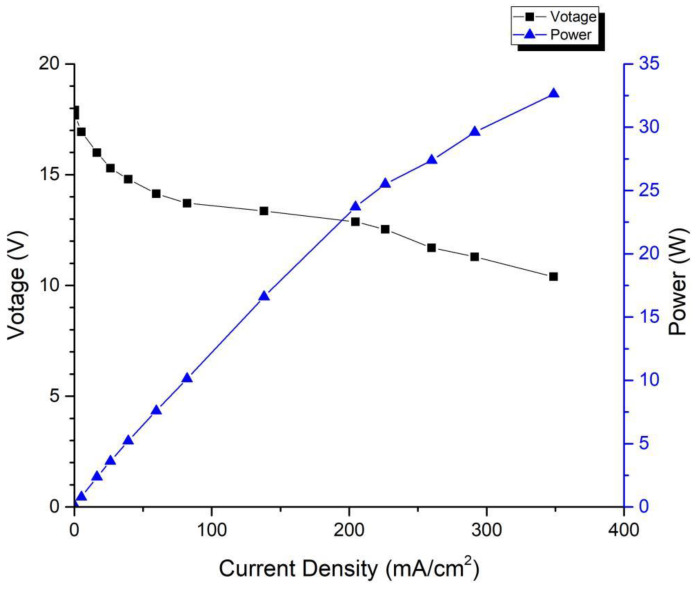
Performance curve of first-generation fuel cell stack.

The test conditions of the fuel cell stack are as follows: the anode is 100% humidified, and the cathode is heated by a fan to force air circulation, which causes the temperature of the cell to fluctuate within ±15 °C. Figure 5 shows the performance curve of the first-generation fuel cell stack, from which it can be observed that a single cell achieves a maximum power of 32.62 W at a voltage of 0.52 V, and a continuous power output of 14.76 W is achievable at a stable voltage of 0.6 V.

Table 1 presents a performance comparison of the open-cathode proton exchange membrane fuel cells currently available. It is evident from the table that the stack designed in this study has not yet achieved leading performance. The reasons for this are as follows: Firstly, activation is an important step before fuel cell operation, and due to the lack of activation, optimal performance cannot be achieved. Secondly, there is room for improvement in the structural design, such as replacing the flow field plates with multiple serpentine flow channel structures. Lastly, by implementing forced convection and cycling the cathode exhaust air, the moisture carried away on the cell membrane material can be recirculated through the system for secondary utilization.

**Figure 6:**
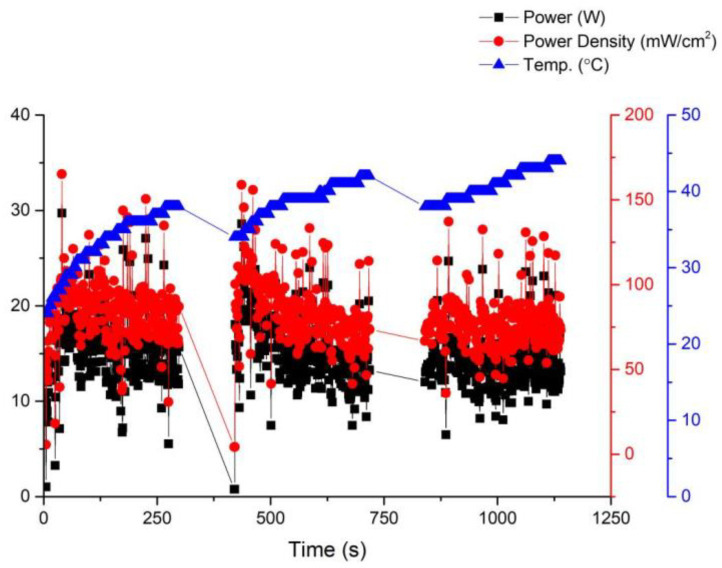
Activation curve of the first version fuel cell stack.

Fuel cell activation is an essential step before its official use. In this paper, fuel cell activation is carried out using a fixed voltage of 14 V, with the anode being 100% humidified and a fan being used for forced cooling. Figure 6 shows in detail the relationship between the performance curve and the temperature of the battery during the activation process. At a working voltage of 0.6 V, the power of the battery can be roughly stabilized at 14 W, indicating that the optimal working temperature of the battery is 35 °C. On the other hand, after running under these conditions for a period of time, it was discovered that the membrane electrode assembly, due to its open-cathode structure, loses moisture and heat generated by the membrane material when forced convection is applied through a fan. This results in a reduction in the membrane material humidity and subsequently affects fuel cell performance. Without forced cooling from a fan, the membrane material would heat up, leading to reduced humidity and hindering further improvement in fuel cell performance.

**Figure 7:**
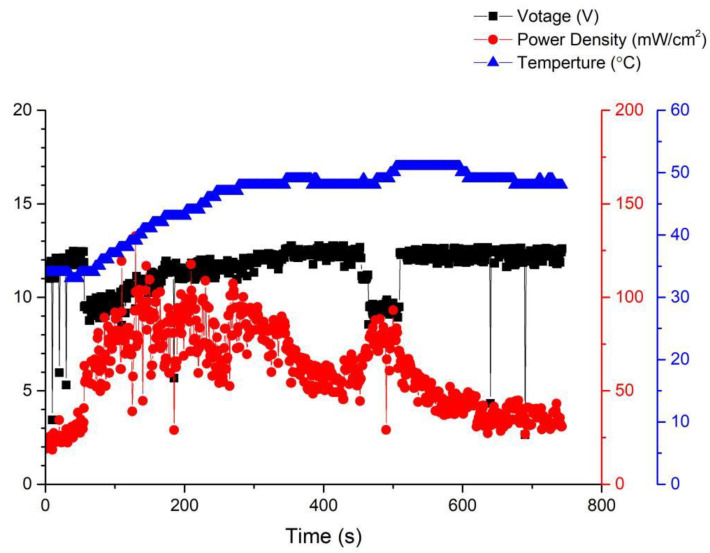
Temperature performance and time curve of the first version fuel cell.

After activation, a gas flow test was conducted on the fuel cell. The anode was set to 100% humidification, and the operating temperature was set to 35 °C. As time went on, the amount of gas introduced into the fuel cell gradually increased, but as seen in Figure 7, it was found that the performance of the fuel cell decreased at all times except for the initial and intermediate stages, where there was an improvement. This is because in the initial situation, as the gas rate increases, it reacts more fully and its performance improves. However, when the gas rate is too large, the excess gas is rushed out of the flow channel before it can react, resulting in a decrease in performance.

#### 3.3.2. Second Version Performance Analysis

Through the testing conducted in Section 3.3.1, it was determined that the performance of the air-cooled stack in this initial version was unsatisfactory. Consequently, based on these findings, the present study proceeded to develop a second version. In the first modification of the second version, two additional fastening bolts were symmetrically introduced to the original design. This enhancement resulted in an improved compression tightness of the membrane electrode assembly and contributed to the overall stability of the fuel cell’s performance [24,25,26]. Furthermore, in the second modification, the air intake method was modified to rear intake. This adjustment is particularly significant when implementing this design in larger stacks as it helps mitigate excessive internal pressure within the fuel cell. Such pressure, if left unaddressed, could potentially lead to gas breakthrough within the membrane electrode assembly and subsequently result in gas leakage [27]. Finally, given that the reaction area of the first version of the membrane electrode assembly was 9 cm^2^, this version has increased by 2.4 times to 21.56 cm^2^ based on the former. Compared with first version, the fuel cell area has increased by about 2.4 times. Two triple-cell stacks of the second version were produced, and different carbon papers were used for the cathodes of the two stacks for comparative testing.

**Figure 8:**
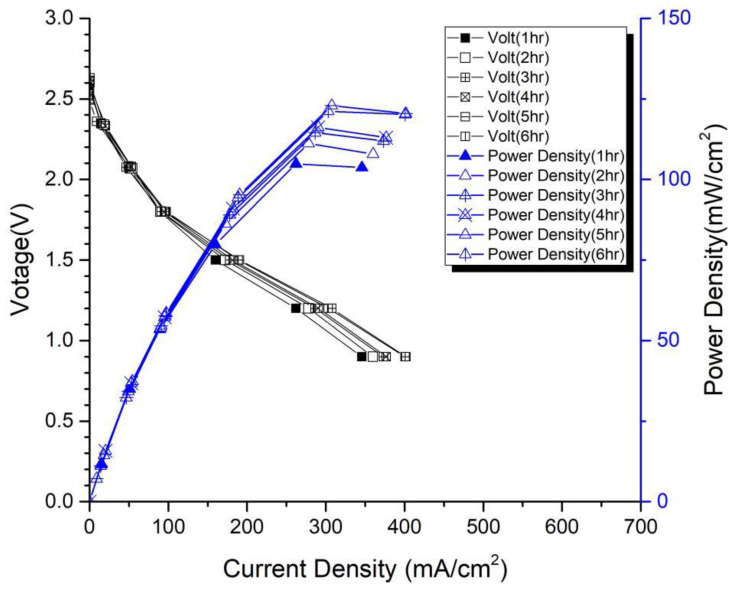
Carbon-Energy-240-activated performance curve for 6 h.

**Figure 9:**
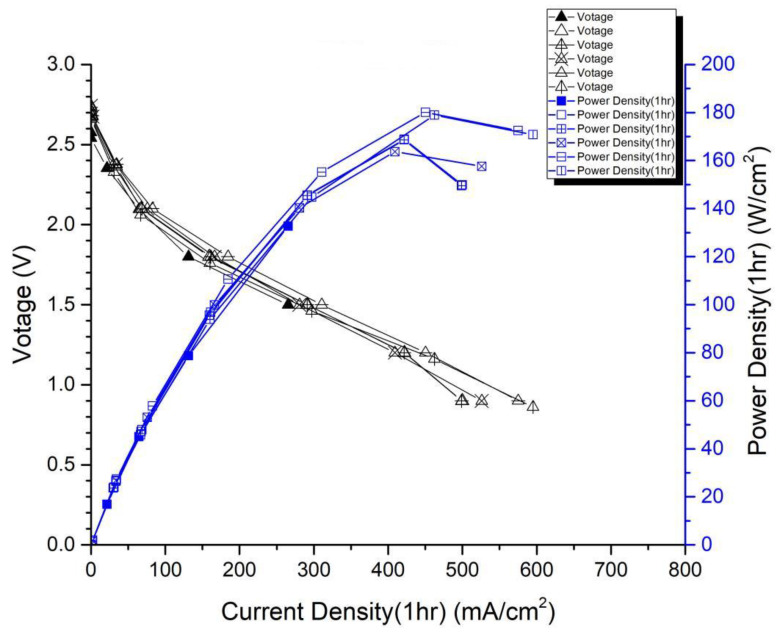
MMPL-activated performance curve for 6 h.

In the first version of the fuel cell stack, due to the absence of external temperature-controlled fans and cathode humidification channels, the activation of the cells was difficult to achieve completely. In the second version of the fuel cell stack, a self-designed cathode humidification channel [28,29] and fan module were incorporated, enabling the activation process of both the cathode and anode of the cell to take place under 100% humidification conditions. This experiment followed the activation guidelines from Taiwan’s Yangzhi Fuel Cell Company: running for 6 h at 0.4 V, with performance testing conducted every hour. Figure 8 and Figure 9 show the performance curves of carbon energy 240 and MMPL after 6 h of activation, respectively. It is found that (1) from the perspective of a certain type of carbon paper alone, the improvement in stack performance is not significant with the passage of activation time. (2) Compared to carbon energy 240, MMPL significantly improves the performance of the stack. This is because MMPL improves the water retention of the fuel cell and increases the uniformity of gas distribution [30,31,32]. (3) The cathode humidification channel and fan module designed in this study addressed the drawbacks of cathode humidification and temperature control in the first version.

From the previous text, it can be deduced that two different fuel cell stacks were fabricated in the second version: one utilizing Carbon Energy 240 carbon paper, and the other using MMPL homemade carbon paper developed in this study. As shown in Table 2, it can be observed that after 6 h of activation, regardless of the operating conditions at 0.6 V or 0.4 V, MMPL outperforms Carbon Energy 240. Under the 0.6 V operating condition, MMPL’s performance surpasses that of Carbon Energy 240 by about 41%, and under the 0.4 V operating condition, MMPL’s performance is approximately 29% better than Carbon Energy 240. Hence, it is evident that MMPL is better suited for an open-cathode fuel cell stack.

**Figure 10:**
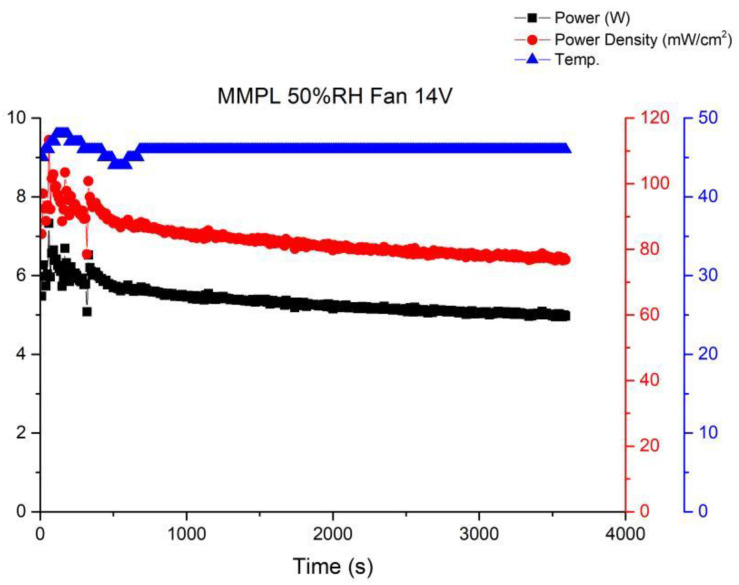
MMPL anode, 50 h, fan—14 V & 1 HR.

Long-term performance testing is the foundation for fuel cell commercial applications. Therefore, this article will continue to investigate the stability of open-cathode fuel cell stacks under long-term operating conditions. Among these, the most important factors are the interaction of cathode gas flow rate, fan module, and temperature. When there is insufficient cathode flow, the membrane electrode assembly cannot fully perform. However, once the cathode airflow is increased by the fan, the cathode will lose a significant amount of moisture to the air, leading to a decrease in humidity. Figure 10 shows the performance curve under the conditions of using MMPL material, a fan driven by a 14 V voltage, 50% humidification, and an operating temperature of 46–48 °C. From the graph, it can be seen that when sufficient flow is provided to the cathode end, the fuel cell performance will immediately improve, but it will subsequently fluctuate and decrease to an equilibrium state. Figure 10 illustrates the performance curve obtained when employing MMPL material, with the fan driven by a 14 V voltage source, maintaining a 50% humidification level, and an operational temperature range of 46–48 °C. The graph clearly depicts that the provision of an ample cathode flow leads to an immediate enhancement in fuel cell performance. However, it subsequently exhibits fluctuations before settling into an equilibrium state characterized by diminished performance levels.

**Figure 11:**
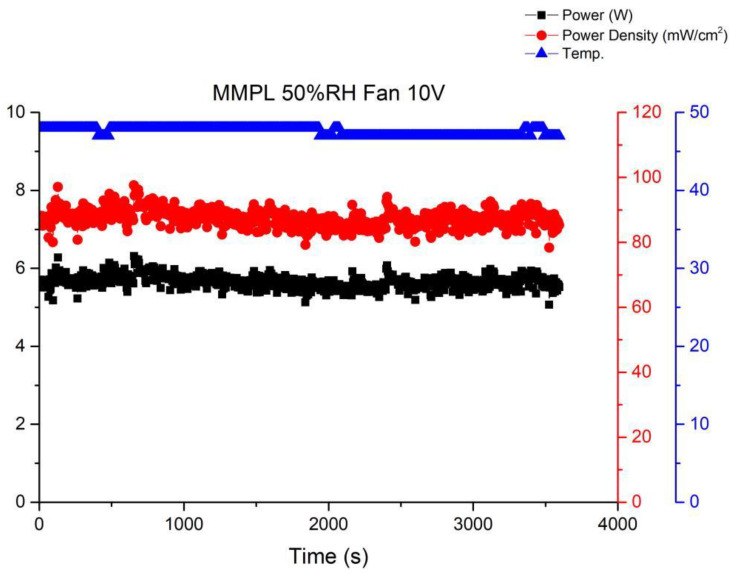
MMPL anode, 50 h, fan—10 V & 1 HR.

Figure 11 illustrates the performance curve subsequent to reducing the fan drive voltage to 10 V. A noticeable uptick in performance fluctuations within the fuel cell stack becomes evident. This phenomenon stems from inadequate gas flow at the cathode of the stack under this specific operational condition, resulting in amplified performance fluctuations. Elevating the flow rate tends to stabilize the fuel cell performance, as depicted in Figure 10. Nevertheless, a high flow rate can lead to a reduction in the moisture level within the stack, ultimately precipitating a decline in the performance of the membrane electrode assembly. Thus, it becomes imperative to ascertain an equilibrium point that ensures fuel cell stability without allowing excessive fan airflow to desiccate the membrane electrode assembly, thereby causing performance degradation.

**Figure 12:**
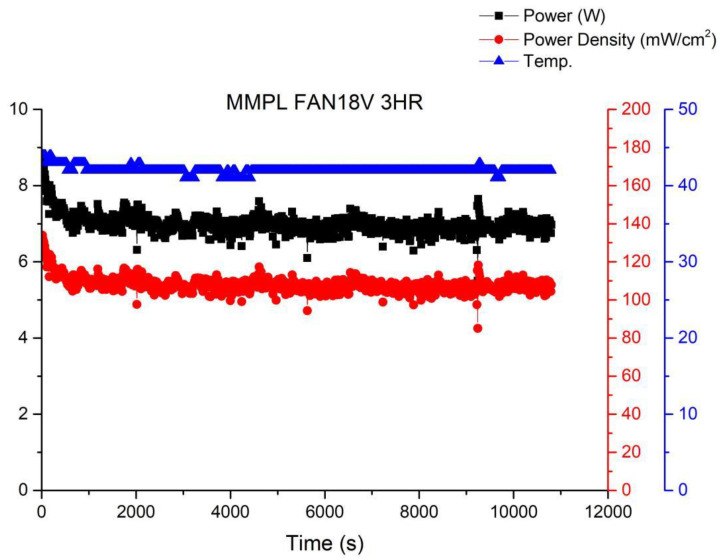
MMPL anode, 50 h, fan—18 V & 3 HR.

Figure 12 shows the operational curve of a fuel cell running for 3 h under the condition of a fan operating at 18 V and a voltage of 0.6 V. It can be observed from the graph that as the fan power increases, the fuel cell performance gradually improves, although fluctuations still persist. This is because with the increase in gas flow rate due to the enhanced fan power, there is sufficient reactant available, preventing a decrease in humidity and subsequent performance degradation caused by excessive gas.

**Figure 13:**
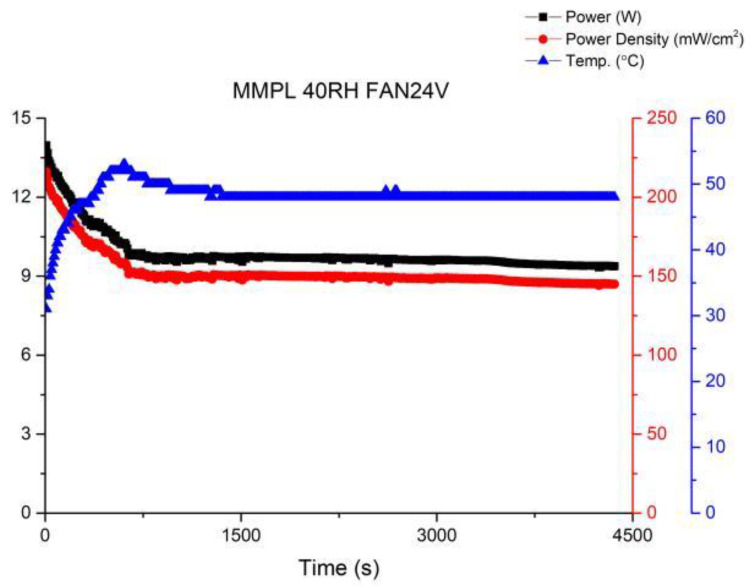
MMPL anode, 50 h, fan—24 V & 1 HR.

In order to further enhance the stability of fuel cell operations, this study attempted to increase the fan voltage to 24 V. The performance curve is shown in Figure 13. It was observed that while the performance steadily improved, the fluctuations were effectively suppressed. This indicates that the equilibrium point for fuel cell operation has been identified, resulting in significant progress compared to the first version of the fuel cell stack.

**Figure 14:**
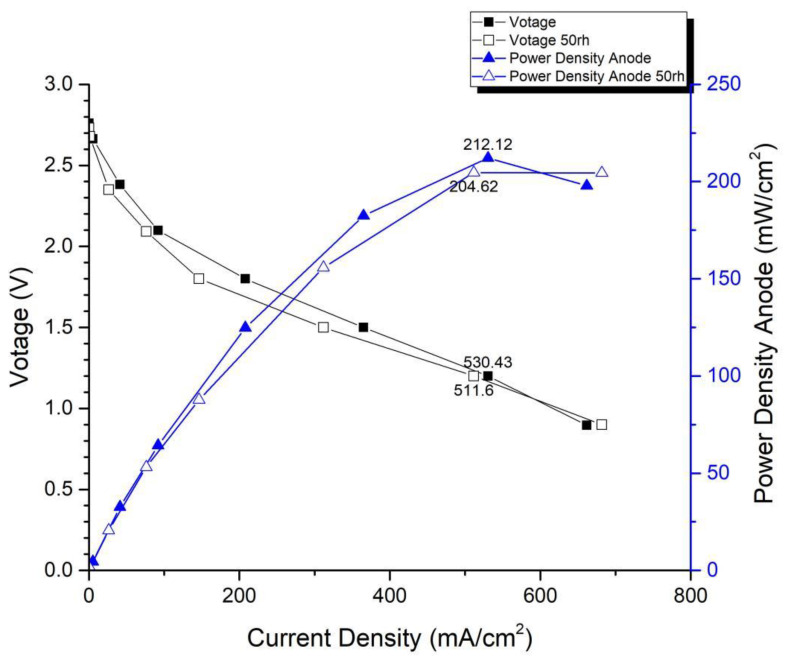
MMPL anode, 50 rh vs. 100 rh.

**Figure 15:**
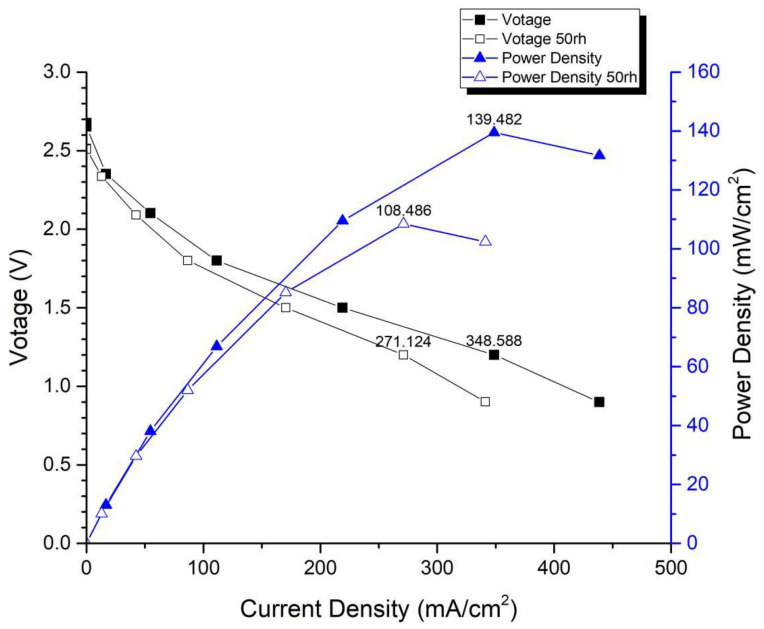
Carbon Energy 240 anode, 50 rh vs. 100 rh.

Figure 14 and Figure 15 depict the performance curves of the second version of the fuel cell stack using Carbon Energy 240 and MMPL under 50% and 100% humidity conditions, respectively. Figure 14 illustrates the test results for MMPL, showing a performance difference of around 10% between 100% and 50% humidification on the anode side under a single-cell voltage of 0.6 V, while the difference is negligible under a single-cell voltage of 0.4 V. Figure 15 presents the test results for Carbon Energy 240, indicating a performance difference of approximately 20% between 100% and 50% humidification on the anode side under a single-cell voltage of 0.6 V. From this, it can be concluded that under typical operating conditions, MMPL’s effective humidification capability mitigates the impact of humidity reduction on fuel cell performance. Consequently, compared to Carbon Energy 240, MMPL demonstrates superior performance.

## 4. Conclusions

Open-cathode fuel cells offer the advantages of low cost and lightweight, reducing the complexity of power systems. Researchers aim to employ them in weight-restricted domains, such as aerospace vehicles. This study yielded the following conclusions:

(1) An open-cathode fuel cell stack was fabricated. To mitigate the cost impact of expensive catalysts, this study utilized nanotechnology to convert platinum into nanoparticles, which were then sprayed onto the membrane material, thereby increasing catalyst utilization. On the other hand, introducing a microporous structure on the membrane material surface not only enhanced the cell’s performance and durability but also addressed water management issues in the cell.

(2) The research found that the three most crucial factors affecting the long-term operational stability of an open-cathode fuel cell stack are cathode gas flow rate, fan speed, and external cooling. Only when these three parameters are optimally coordinated can the fuel cell achieve its best performance. Firstly, when the cathode flow rate is insufficient, the fuel cell cannot fully realize its performance potential. Secondly, simply increasing the fan speed to boost the gas flow rate can reduce the internal humidity of the cell, leading to a decrease in performance. This study, after conducting system experiments, has determined that using MMPL carbon paper and driving the fan at 24 V voltage can ensure an adequate gas flow rate and maintain the cell’s humidity at around 50%, allowing the fuel cell to achieve optimal performance output.

## Figures and Tables

**Table 1:** Performance comparison of different types of fuel cell stacks.

Open-Cathode Stack	Stack Picture	Power/Power Density	Weight/Volume	Cell No./MEA Size	Design Concept and Materials
This paper	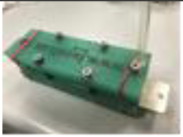	13.474 W/208 mW/cm^2^@0.4 V	~370 g	3/21.56 cm^2^	For UAV
C.Y. Ling [20]	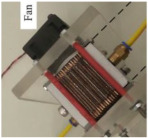	120 W/348 mW/cm^2^@0.6	~329 g/65 × 75 × 45 mm	15/23 cm^2^	Dupont MEA3
Zhen-Ming Huang [21]	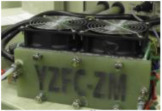	321 W/178 mW/cm^2^@0.58 V	5700 g/140 × 258 × 100 mm	18/100 cm^2^	Nanya MEA
Houchang Pei [22]	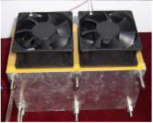	560 W/295 mW/cm^2^@0.42 V	-	19/100 cm^2^	Optimal GDL, WHUTMEA, Nafion211
Santa Rosa [23]	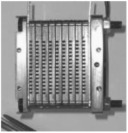	9.4 W/310 mW/cm^2^@0.39 V	-	8/3.8 cm^2^	3M MEA

**Table 2:** Performance comparison of different carbon papers in fuel cells.

	Current Density@0.6 V (mA)	Power@0.6 V (W)	Power Density@0.6 V (mW)
Carbon Energy 240	97.509	3.784	58.504
MMPL	166.54	6.4624	99.914
MMPL compared to Carbon Energy 240	41%	41%	41%
	**Current Density@0.4 V**	**Power@0.4 V**	**Power Density@0.4 V**
Carbon Energy 240	307.38	7.9517	122.94
MMPL	430.38	11.136	172.18
compared to Carbon Energy 240	28%	28%	28%

## Data Availability

The data presented in this study are available on request from the corresponding author.
